# Distinct serum apolipoprotein A-I levels in neuromyelitis optica and acute transverse myelitis

**DOI:** 10.1186/1476-511X-12-150

**Published:** 2013-10-23

**Authors:** Yu-Hua Zhong, Jia Liu, Min Li, Xuan Wang, Yuan Yuan, Xiu-Feng Zhong, Fu-Hua Peng

**Affiliations:** 1 Multiple Sclerosis Center, Department of Neurology, The Third Affiliated Hospital of Sun Yat-Sen University, 600 Tianhe Road, Guangzhou, Guangdong Province 510630, China; 2 Wilmer Eye Institute, Johns Hopkins University School of Medicine, 400 N.Broadway, Baltimore, MD 21093, USA

**Keywords:** Apolipoprotein(apo) A-I, Neuromyelitis optica, Acute ransverse myelitis

## Abstract

**Objective:**

NMO and ATM are intertwined both clinically and pathologically. Apolipoprotein (apo) A-I, the main apolipoprotein of HDL, plays an important role in lipid metabolism in the cerebrospinal fluid and is known to suppress pro-inflammatory cytokines generated by activated T cells in some autoimmune diseases as an immune regulator. However, the differences in the levels of serum apoA-I between NMO and ATM patients are unclear.

**Methods:**

In the present study, serum apo A-I levels were measured in 53 NMO patients, 45 ATM patients and 49 healthy subjects. We tested serum apoA-I levels in all participants and investigated EDSS scores of patients with NMO and ATM. Statistical analyses were performed by using SPSS statistical software.

**Result:**

We found that serum apoA-I levels in patients with NMO were significantly lower in comparison to those with ATM. We also found that serum levels of apoA-I was lower in male subjects in comparison to the female subjects in all groups although these differences were not statistically significant in patients with NMO or ATM. It is also shown in our study that serum apoA-I levels in patients with NMO were significantly elevated after receiving a high dosage of intravenous corticosteroids over a period of one week. However, we did not find any correlation between the apoA-I levels and disease disability.

**Conclusion:**

From this study, we concluded that serum levels of apoA-I were lower in NMO patients compared to patients with ATM. Serum apoA-I studies might provide some useful clues to differentiate NMO cases from ATM cases.

## Introduction

Neuromyelitis optica (NMO), as a rare autoimmune demyelinateting disorder, arouses inflammatory lesions in the optic nerves and spinal cord and causes some serious clinical symptoms such as blindness and paralysis. In NMO, the inflammatory profile primarily involves eosinophils/neutrophils and autoantibody reaction. At present, an autoantibody (NMO IgG) against aquaporin-4 (AQP4), a water channel expressed on astrocytes, has been incriminated as a causative factor. Similar to other autoimmune diseases, Th17 cells and their effective cytokines (such as interleukin 6) participate in the pathogenesis of NMO [1,2]. Acute transverse myelitis (ATM) is characterized as a demyelinating, inflammatory, and infectious myelopathy, with a variety of clinical manifestations, including those associated with multiple sclerosis (MS) [3], NMO [4], systemic autoimmune disease [5], infection [6], as well as cases with no specific origin(idiopathic ATM) [7]. Although acute transverse myelopathy can be presented in a variety of ways and involves pyramidal, sensory, and autonomic dysfunction to varying degrees, the signs and symptoms of myelopathy do not provide an insight into the etiology and the differential diagnosis of the disease. Therefore, there is a requirement for potential-molecular markers to differentiate ATM from other demyelinating and inflammatory myelopathies, including MS, NMO, other systemic inflammatory diseases (SLE), acute disseminated encephalomyelitis, and postvaccinial myelitis [8]. This article emphasizes the differential diagnoses between NMO and ATM. Despite the reported differences, NMO and ATM are still intertwined both clinically and pathologically. In addition, ATM can be the first manifestation of MS and NMO. Thus, it will be very useful if plasma-based biomarkers can be identified to discriminate NMO from ATM.

Apolipoprotein (apo)A-I, as the main component of high density lipoprotein (HDL), plays a vital role in reverse cholesterol transportation by facilitating the binding of HDL and lecithin cholesterol acyltransferase [9]. The central nervous system is the most lipid-rich organ and approximately 25% of the total body’ cholesterol is distributed in the central nervous system [10]. It has previously been shown that apoA-I has been implicated in several antiatherogenic functions, including protection against thrombosis and oxidative stress [11]. Beyond that, apoA-I can protect hippocampal neuronal cultures from amyloid beta-induced neurotoxicity as well [12]. Moreover, apoA-I might play the role of a constitutive anti-inflammatory factor [13]. Therefore, the aim of this study was to evaluate the differences in serum apoA-I levels between patients with NMO and ATM.

### Patients and methods

Serum samples were collected from 147 individuals who had been treated from January 1, 2006 to December 31, 2012 in the Third Affiliated Hospital of Sun Yat-Sen University, Guangzhou, China. These patientscomprised of 53 patients with NMO, 45 patients with ATM and 49 healthy subjects. Demographic and EDSS scores of NMO and ATM patients and healthy control (HC) group were presented in Table 1. All NMO patients were diagnosed with NMO according to the diagnostic criteria in 1999 [14] and had been in hospital for the first onset (n = 40) or acute relapse (n =13). All the ATM patients were included according to a modified version of the Transverse Myelitis Consortium Working Group criteria [15]. 45 patients were with a first episode of ATM and had excluded a compressive etiology. All patients were scored by the Expanded Disability Status Scale (EDSS). The mean EDSS score was 4.6 ± 2.0 in NMO patients and 5.0 ± 2.7 in ATM patients.

**Table 1:** Demographic and clinical characteristics of NMO, ATM patients and healthy control group

**Patients**	**NMO (n = 53)**	**ATM (n = 45)**	**HC (n = 49)**
Male	17	29	25
Female	36	16	24
Range of ages (y)	8–70	5–66	9–70
Means of ages (y)	38.2	39.0	41.0
EDSS	4.6 ± 2.0	5.0 ± 2.7	

All selected patients had never received steroids or statins treatments or disease-modifying immunosuppressive therapy that can affect the serum apoA-I levels two months before admission. The blood was collected to detect serum apoA-I after overnight fasting. Serum apoA-I levels were measured by routine clinical testing. This study was approved by the Ethics Committee of the Third Affiliated Hospital of Sun Yat-Sen University (200733) and in compliance with the Helsinki Declaration. All subjects gave informed consents to participate in this study.

### Statistical analysis

All statistical analyses were performed using the Statistical Program for Social Sciences (SPSS) statistical software (version 11.0, Chicago, IL, USA). All the data in this study were presented as means ± SD. Statistical significance was set at P < 0.05. The effect of age on serum apoA-I levels of different groups were analyzed by covariance analysis. The comparison of serum apoA-I levels among the NMO, ATM patients and the HC group was performed using covariance analysis with age as the covariant. Since serum apoA-I levels can be moderated by estrogen levels, the patients were divided into two subgroups according to the gender. Covariance analysis was also used to compare serum apoA-I levels of males and females patients with NMO, ATM or in HC group with age as the covariant. Correlations between serum apoA-I and EDSS scores of NMO and ATM patients were analyzed by Spearman correlation analysis.

## Results

In this study, significant differences were found in the levels of serum apoA-I, depending on the disease type and gender. Firstly, apoA-I levels in different disease groups were compared without consideration of gender and age factors. The results showed that the serum apoA-I levels in NMO (1.20 ± 0.21 g/L) and ATM (1.32 ± 0.22 g/L) disease were significantly lower than the levels measured for healthy controls (1.59 ± 0.13 g/L). Patients with NMO (1.20 ± 0.21 g/L) had significantly lower serum apoA-I levels in comparison to patients with ATM (1.32 ± 0.22 g/L) (Table 2).

**Table 2:** Analysis of serum apoA-I among the patients with NMO and ATM and healthy control

**Group**	**Gender ratio (female: male)**	**Means age ± SD (years)**	**ApoA-I (g/L)**	**p1**	**p2**	**p3**
NMO	36:17	38.2 ± 14.4	1.20 ± 0.21	0.006^*^		
ATM	16:29	39.0 ± 16.1	1.32 ± 0.22		<0.001^**^	
HC	24:25	41.0 ± 17.7	1.59 ± 0.13			<0.001^**^

Data are means ± SD. p1, NMO versus ATM; p2, ATM versus HC; p3, NMO versus HC.

*Difference is significant below the 0.01 level.

**Difference is significant below the 0.001 level

**Figure 1:**
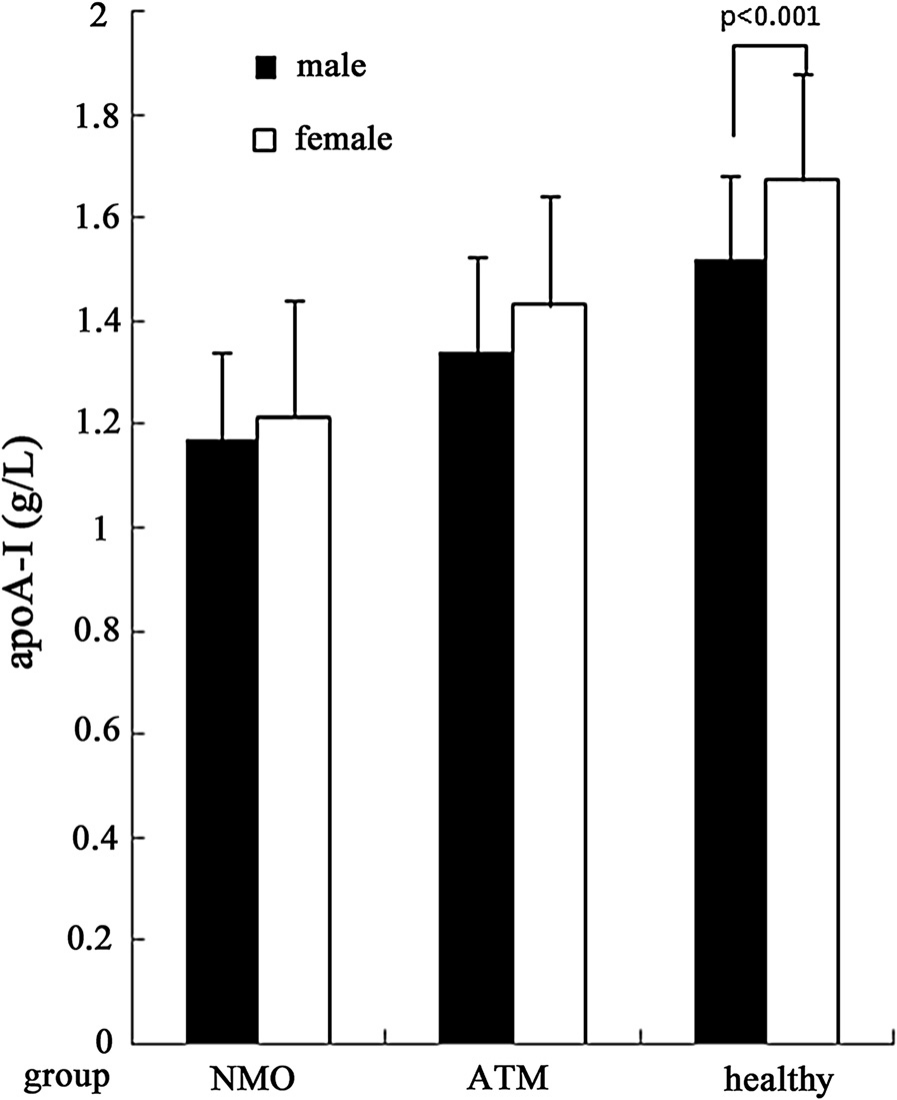
Gender influence on serum apoA-I levels within each group.

**Figure 2:**
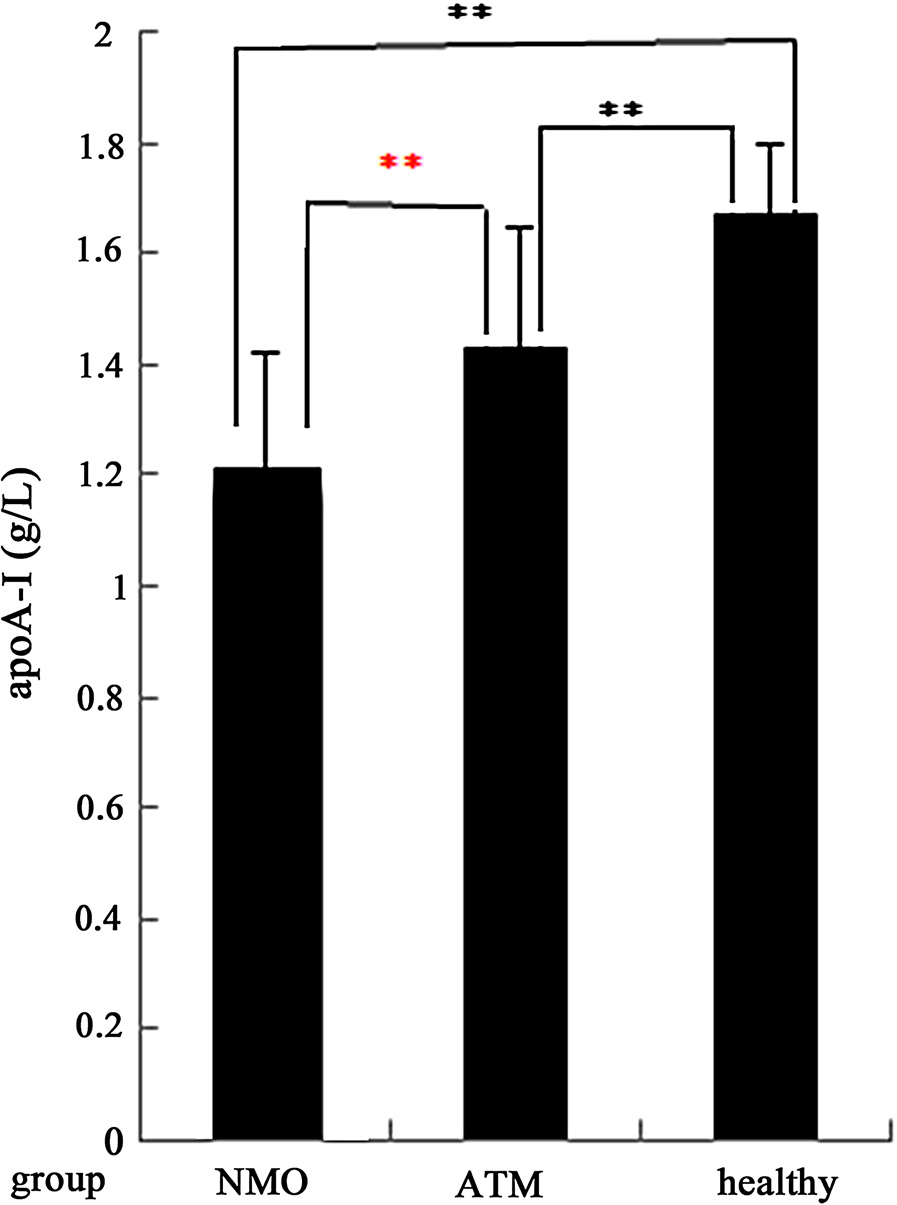
**Comparison of serum apoA-I levels in female among NMO, ATM patients and healthy control.** Black star: HC versus NMO or ATM; red star: NMO versus ATM. **: p < 0.001.

**Figure 3:**
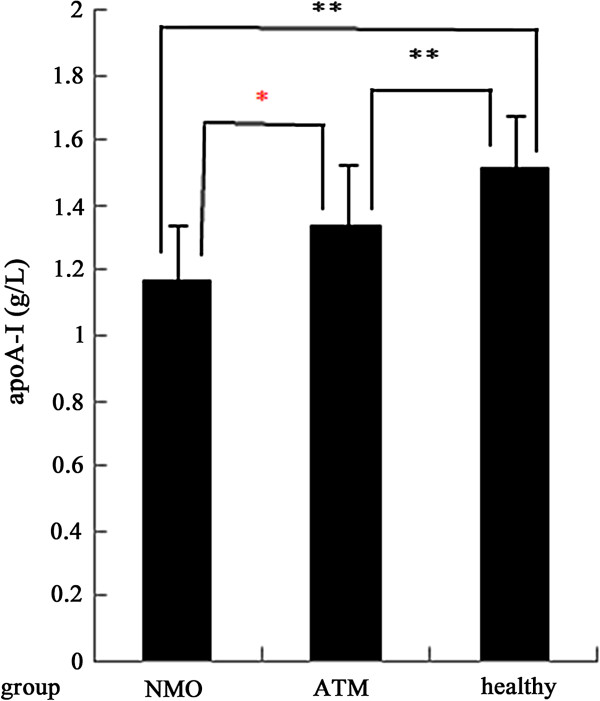
**Comparison of serum apoA-I levels in male among NMO, ATM patients and healthy control.** Black star: HC versus NMO or ATM ; red star: NMO versus ATM. *: p < 0.01; **: p <0.001.

In order to assess the influences of gender on serum apoA-I levels, we made a comparison between male and female patients on serum apoA-I in each disease group and healthy group. For NMO and ATM patients, male patients had lower apoA-I levels than female patients though the differences were not statistically significant. The serum apoA-I levels were much higher in female HC group (1.67 ± 0.21 g/L) than in the corresponding male HC control (1.52 ± 0.16 g/L) (p < 0.001) (Figure 1). Furthermore, we compared with male and female patients respectively (Table 3). The results suggested that female NMO patients (1.21 ± 0.23 g/L) had the lowest serum apoA-I level and female HC group (1.67 ± 0.21 g/L) had the highest serum apoA-I levels (Figure 2). Female NMO patients also had significantly lower serum apoA-I levels than female ATM patients (p = 0.001). Similarly, Male patients with NMO had significantly lower serum apoA-I levels than male patients with ATM (p = 0.002) (Figure 3). The serum apoA-I levels of male patients in both NMO and ATM groups were significantly lower than those in the the healthy controls. To sum up, serum apoA-I levels was lowest in male NMO patients and highest in female HC group in this study.

**Table 3:** Serum apoA-I levels in male and female NMO, ATM patients and healthy controls

	**Female**	**ApoA-I (g/L)**	**Male**	**ApoA-I(g/L)**	**p1**	**p2**	**p3**
	**Age**		**Age**				
NMO	38.1 ± 13.2	1.21 ± 0.23	38.5 ± 17.3	1.17 ± 0.17	0.001^*a^	0.002^*a^	0.471
ATM	39.8 ± 16.1	1.43 ± 0.22	38.6 ± 16.4	1.34 ± 0.19	0.001*^b^	<0.001^**b^	0.056
HC	39.3 ± 18.7	1.67 ± 0.21	42.5 ± 16.8	1.52 ± 0.16	<0.001^**c^	<0.001^**c^	<0.001^**^

Data are means ± SD. p1, Compared serum apoA-I levels among three female groups; p2, Compared serum apoA-I levels among three male groups; ^a^NMO versus ATM; ^b^ATM versus HC; ^c^NMO versus HC; p3, Compared serum apoA-I levels between female and male NMO patients, and female and male patients in other two groups.

*Difference is significant below the 0.01 level.

**Difference is significant below the 0.001 level.

**Figure 4:**
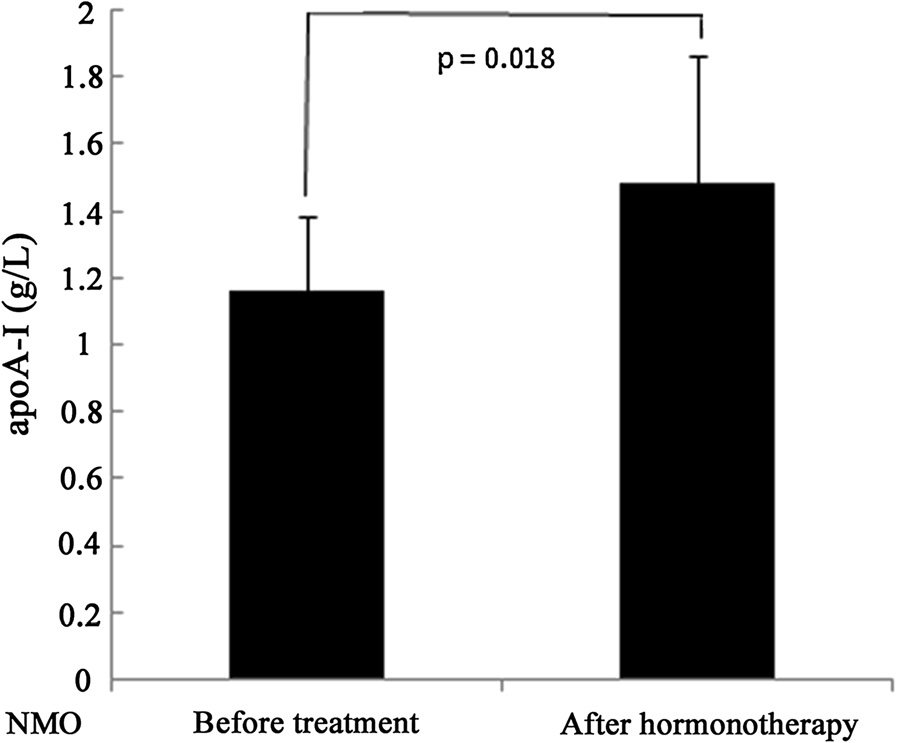
**Influence of corticosteroid treatment on serum apoA-I levels of patients with NMO.** High-dose intravenous corticosteroid treatment was given to patients with NMO for one week.

Finally, we explored the correlation between apoA-I levels and EDSS scores of patients with NMO and ATM and the changes of serum apoA-I levels in NMO patients who received a high dose intravenous corticosteroids treatment for one week. We found a negative correlation between the apoA-I levels and EDSS scores in NMO patients although the correlations showed no statistical differences. Serum apoA-I levels were significantly elevated after receiving high dosage of intravenous corticosteroids for one week (Figure 4).

## Discussion

Our research showed that the serum apoA-I levels in NMO and ATM were significantly lower than those in healthy group. Although the exact reason for these differences remained unknown, the following evidences may explain this phenomenon. It is well-known that multiple factors are involved in the development of their pathological process, such as genetic susceptibility, gender and environment. The autoimmune-inflammation reaction was believed to play a critical role in such process. The immune-inflammation reaction was characterized by the infiltration of immune-inflammatory cells and plasma factors into the lesion site to eradicate infection and to repair damaged tissues [13]. Contact-mediated activation of monocytes by stimulated T lymphocytes acted as a major stimulus, triggering the production of large amounts of tumor necrosis factor-α (TNF-α) and interleukin-1β (IL-1 β) which played an important role in chronic inflammation [16]. ApoA-I, as a specific inhibitor of cytokine production in monocyte-macrophages upon contact with stimulated T cells, exerted most of its inhibitory activity by specifically binding the activating factor on stimulated T cells, consequently blocking the interaction between T-lymphocytes and monocytes [17]. Therefore, apoA-I was known as a negative acute-phase protein and might diffuse into the extra-vascular compartment and interfere with the cross-talk between cells in acute or chronic inflammation [16], resulting in a decrease of serum apoA-I levels. In addition, the maintenance of inflammation in a particular target tissue would in turn keep up a low concentration of apoA-I at the systemic level and thus be beneficial to the production of inflammatory cytokines [18]. This could account for a significantly lower serum apoA-I levels in NMO and ATM patients in comparison to the corresponding serum apoA-I levels in the healthy group. This also implied that apoA-I may be a sign of the possible development of inflammation. ApoA-I level was decreased in the plasma of rheumatoid arthritis (RA) patients. However, it was enhanced in synovial fluid of RA patients. It has been reported that ApoA-I also exists in human cerebrospinal fluid [19]. Therefore, we need to explore the changes of CSF apoA-I levels in NMO and ATM in the future.

In this study, another notable finding was the significantly lower serum apoA-I levels in patients with NMO in comparison to those with ATM. Although the reason is still unclear, it may be associated with the following factors. Several reports indicated that humoral immunity participated in the pathogenesis of NMO [20]. The discovery of NMO IgG, which is directed against aquaporin-4 (AQP4), is the strong evidence. A subset of B-cells in the peripheral tissues is stimulated to produce anti-AQP4 IgG antibody, which results in an extensive loss of astrocytes in specific regions of the CNS via complement mediated cytotoxicity [1]. It had been previously confirmed that astrocytes could generate apoA-I in rat [21], and the extensive loss of astrocytes resulted in significantly decreased productions of apoA-I. Moreover, some studies indicated that apoA-I plasma concentrations was diminished in SLE and this decrease was associated with the presence of anti-apoA-I antibodies in 32% of patients [22]. Such specific anti-apoA-I antibodies are likely to exist in NMO patients because humoral immunity involved in the pathological process. In addition, based on a survey of serum cytokine levels [23], the study indicated that increased inflammation existed in patients with NMO compared to patients with MS. Therefore, we cannot excludethe possibility that patients with NMO have an increased inflammatory response in comparison to patients with ATM, resulting in the increased utilization of apoA-I. Based on the above mentioned reasons, it is reasonable that patients with NMO might have significantly lower serum apoA-I levels than the patients with ATM as shown in our results. Our results also suggest that the serum apoA-I levels in NMO might provide some useful clues for the differential diagnosis against ATM.

Finally, we found that the serum levels of apoA-I were lower in males than in females in all the analyzed groups, though the differences were not statistically significant in patients with NMO or ATM. It could be explained by that apoA-I levels are moderated by estrogen levels [24]. However, a correlation between apoA-I and EDSS score in NMO and ATM patients showed no statistical differences. This suggested that apoA-I level was not correlated with disease disability. However, further researches are needed to verify this finding with a larger patient population. Interestingly, the serum ApoA-I levels were significantly increased after NMO patients received corticosteroid treatment for one week. Corticosteroids have anti-inflammatory properties and immunosuppressive effects that could result in decrease of the productions of cytokines and stimulated T lymphocytes [4], resulting in increased levels of apoA-I after corticosteroid administration.

In the future, we plan to measure the apoA-I levels in cerebrospinal fluid of the patients suffered from NMO and ATM and discuss the relationship between CSF apoA-I and clinical presentations of NMO and ATM patients. We also intend to collect more study subjects to seek out more clues to help differentiate these two diseases at outset.

## Competing interests

The authors declare that they have no competing interests.

## Authors’ contributions

YZ collected and analyzed data and drafted the manuscript; JL and ML carried out analytical and statistical; YY and XW carried out analytical work; XZ and FP, co-designed the study, FP interpreted data and wrote the manuscript. All authors read and approved the final manuscript.
